# Deep Reinforcement Learning for Resource Management on Network Slicing: A Survey

**DOI:** 10.3390/s22083031

**Published:** 2022-04-15

**Authors:** Johanna Andrea Hurtado Sánchez, Katherine Casilimas, Oscar Mauricio Caicedo Rendon

**Affiliations:** Departamento de Telemática, Universidad del Cauca, Popayan 190002, Colombia; johannahurtado@unicauca.edu.co (J.A.H.S.); lkcasilimas@unicauca.edu.co (K.C.)

**Keywords:** admission control, resource allocation, resource scheduling, resource orchestration, network slicing, deep reinforcement learning

## Abstract

Network Slicing and Deep Reinforcement Learning (DRL) are vital enablers for achieving 5G and 6G networks. A 5G/6G network can comprise various network slices from unique or multiple tenants. Network providers need to perform intelligent and efficient resource management to offer slices that meet the quality of service and quality of experience requirements of 5G/6G use cases. Resource management is far from being a straightforward task. This task demands complex and dynamic mechanisms to control admission and allocate, schedule, and orchestrate resources. Intelligent and effective resource management needs to predict the services’ demand coming from tenants (each tenant with multiple network slice requests) and achieve autonomous behavior of slices. This paper identifies the relevant phases for resource management in network slicing and analyzes approaches using reinforcement learning (RL) and DRL algorithms for realizing each phase autonomously. We analyze the approaches according to the optimization objective, the network focus (core, radio access, edge, and end-to-end network), the space of states, the space of actions, the algorithms, the structure of deep neural networks, the exploration–exploitation method, and the use cases (or vertical applications). We also provide research directions related to RL/DRL-based network slice resource management.

## 1. Introduction

A major goal of 5G and 6G networks, from now on called 5G/6G, is to deliver a wide variety of services with distinct performance requirements under a (physical/virtual) shared infrastructure [1]. All 5G/6G networks must offer high-speed connections, very high reliability, and extremely low latency for empowering different verticals and enabling new business models [2,3]. These networks promote realizing novel use cases, including ultrareliable low-latency communication (uRLLC), massive machine-type communication (mMTC), enhanced mobile broadband (eMBB), strengthened enhanced mobile broadband (sEMBB), ultramassive machine-type communications (umMTC), massive ultrareliable low-latency communications (mURLLC), mobile broadband reliable low-latency communications (MBRLLC), and extremely reliable and low-latency communications (ERLLC) [1,4,5,6].

Network slicing (NSL) and deep reinforcement learning (DRL) are two key enabling technologies of 5G/6G [7]. A 5G/6G network can comprise one or more network slices belonging to single or multiple tenants. A slice is a customized and isolated logical network conceived to support strict quality of service (QoS) and quality of experience (QoE) requirements [8,9] such as those demanded by, for instance, remote surgeries and immersive media. Network providers need to perform intelligent and efficient resource management to realize slices that meet the requirements of 5G and 6G use cases. Resource management is far from being a straightforward task since it requires mechanisms for its constitutive phases: admission control (accept/reject multitenant network slice requests—NSLRs) [10], resource allocation (assign resources to admitted NSLRs) [11], resource scheduling (program the timely use of allocated resources) [12], and resource orchestration (instantiate and manage the life cycle of slices) [13]. In each phase, the mechanisms must meet diverse performance requirements (e.g., reliability, throughput, latency, packet loss), while increasing provider profits, improving network utilization, and guaranteeing resource provisioning (or re-provisioning) dynamically [14]. Furthermore, intelligent and effective resource management involves predicting the demand coming from many tenants (each tenant with multiple NSLRs) and achieving autonomous behavior of slices.

Although many studies have proposed solutions to manage resources in NSL using, for instance, different heuristics [15,16,17] and genetic algorithms [18], this paper surveys network slicing resource management approaches based on reinforcement learning (RL) and DRL techniques. RL and DRL will play a critical role in turning 5G/6G into a reality. Remarkably, they allow evolving resource management in NSL from techniques based on models to those without models, which learn deeply by interacting with the environment to satisfy experience level agreements (XLA) related to QoE and service level agreements (SLA) associated with QoS. In RL, an agent makes decisions considering the environment’s states (e.g., set of computing and networking resources available to attend slices). Decisions are made to select actions (e.g., allocate and instantiate a node for an accepted NSLR) to apply in an environment. A RL agent monitors the result (expressed as a reward, for example, optimized network utilization) of its interaction with the environment (e.g., a 5G/6G physical network) to adjust its strategy to achieve an optimal policy automatically (e.g., optimize the action selection to support requirements of MBRLLC slices) [19,20]. RL approaches slowly converge to the optimal policy when exploring and acquiring knowledge in large-state action sets, making it difficult to use in large-scale 5G/6G deployments. Deep learning (DL) has been used to face RL limitations, leading to DRL [21,22]. From a high-abstraction level, DRL uses RL to train deep neural networks (DNNs), such as feed-forward neural networks (FNNs) [23] and recurrent neural networks (RNNs) [24], to quickly learn accurate optimal policies.

Though there are various surveys involving DRL and resource management [14,25,26,27,28,29], this survey is purposefully different. Reference [25] reviews DRL techniques without focusing on the networking domain. Unlike [14], which presents a comprehensive survey on ML for networking, and [27,28,29], which introduces a complete revision on DRL techniques for networking, communication networks, and HetNets, our work focuses on approaches using RL and DRL for realizing resource management in NSL. Reference [26] presents, as we do, a survey on network slicing resource management. However, it does not discuss resource management from the perspective of its constitutive phases (admission, allocation, scheduling, and orchestration), includes only a constrained research directions section, and is outdated (published in 2018). To sum up, in contrast to the existing surveys, this paper addresses the following research questions: (i) What are the phases of network slicing resource management and which RL/DRL-based approaches are useful in each phase?. (ii) What are the research directions on RL/DRL-based network slicing resource management?.

The contributions of this paper are:

- A comprehensive view of RL/DRL-based resource management in NSL. The literature published in peer-reviewed venues over the past four years that have a high impact and have been well received by peers is explored and analyzed from the perspective of the main resource management phase in which each proposed approach operates. In addition, the elements used per the RL/DRL technique are detailed for each approach.
- Identification of challenges and research directions in network slice resource management. The presented discussion on RL/DRL-based resource management in NSL uncovers fundamental research challenges to achieve cognitive and autonomous 5G, 6G, and beyond networks. The discussion motivates performing future work to push the boundaries of cognitive networking.

The rest of this paper is organized as follows. Section 2 presents the methodology used to compile our survey and the fundamental concepts needed to understand it. Section 3, Section 4, Section 5 and Section 6 describe works that use RL and DRL to perform admission control, resource allocation, resource scheduling, and resource orchestration, respectively. Section 7 raises challenges and future research directions on RL/DRL-based resource management in NSL. Section 8 concludes this survey. For the sake of readability, Abbreviations provides the list of acronyms and definitions used in this survey.

## 2. Methodology and Foundations

This section introduces the methodology used to carry out this survey. Furthermore, the fundamental concepts around network slicing, resource management, RL, and DRL are presented briefly.

### 2.1. Methodology

To address the research questions raised in this paper, we initially define the network-slicing resource management process by considering admission control, resource allocation, resource scheduling, and resource orchestration phases. Then, we classify the existing RL/DRL-based resource management approaches into one of the phases mentioned. Lastly, we provide research directions in the RL/DRL-based network slicing resource management area.

To select the works presenting network-slicing resource management approaches based on RL and DRL, we introduced search keywords on three electronic databases: SCOPUS, IEEE, and Web of Science. The keywords used: “Resource management on network slicing”, “(DRL or RL) and network slicing”, “(DRL or RL) and 5G”, and “(DRL or RL) and 5 GB”. Not many publications were found because resource management on network slicing is a relatively new research field. Therefore, an additional search using the following keywords was carried out: “admission control and (DRL or RL) and network slicing”, “resource allocation and (DRL or RL) and network slicing”, “resource Scheduling and (DRL or RL) and network slicing”, and ”resource orchestrating and (DRL or RL) and network slicing”. The searches resulted in 150 works. Titles and abstracts were reviewed to eliminate works with no relation to the area. Furthermore, recent works from nonrecognized conferences and low-impact journals were filtered. As a result, we chose 50 works for a full-text review.

The details of the literature review and the analysis performed is presented in Section 3, Section 4, Section 5 and Section 6. Table 1, Table 2, Table 3, Table 4 and Table 5 summarize the works selected to review; the first four works of each table and Section 3.1, Section 4.1, Section 4.2, Section 5.1 and Section 6.1 correspond to papers published in journals and conferences with the highest impact factor. In those tables, the works are analyzed according to optimization objective, network focus (i.e., E2E, RAN, CN, Edge), space of states, space of actions, RL/DRL algorithm, DNN structure, exploration–exploitation method, use case (or vertical application), training, dataset, and development.

### 2.2. Resource Management in Network Slicing

5G is envisioned as a network to support multiple services with specific performance requirements in highly heterogeneous environments [30,31]. Furthermore, 5G is characterized by supporting multiple types of access technologies and shared infrastructures for minimizing service deployment costs, improving network utilization, and increasing network providers’ revenue [32]. Technologies such as network functions virtualization (NFV), software-defined networking (SDN) [33], and NSL [34] are pivotal for realizing 5G networks. NFV allows accomplishing 5G virtual network functions (VNFs) on virtual machines and containers running on commodity hardware [11]. SDN enables flexible management and a global view of 5G network functions, collecting various network data [35]. NSL permits serving 5G services by end-to-end slices defined as logical networks, mutually isolated on shared infrastructure [9,36,37]. Usually, a slice comprises one or more service chains formed by network functions (virtualized or not) and the (physical/virtual) links connecting them [8].

**Table 1:** Admission control based on RL and DRL.

Ref.	Algorithm	Focus	Optimization Objective	Explore-Exploit	NN Structure	Use Case/Vertical App	Training	Dataset	Development
[38]	N3AC	RAN	Meet service guarantees while maximizing profit	ϵ−greedy	FNN	Elastic and inelastic *			Emulation (Keras-TensorFlow)
[39]	SARSA	E2E (RAN, TN, CN, Edge)	Maximize revenue while minimizing dropping probability of NSLRs	ϵ−greedy	Non Apply	QoS and best effort slices *			Simulation (Undeclared tool)
[40,41]	DQN	RAN & TN	Maximize revenue while minimizing slice degradation	Undeclared	FNN	High and low priority *			Emulation (Python-NetworkX)
[42]	DQN	RAN	Maximize revenue while minimizing costs related to SLA violations	ϵ−greedy	Target NN, Online NN	eMBB, uRLLC, and mMTC	Centralized	Synthetic	Simulation (Undeclared tool)
[43]	Q-learning R-learning	CN	Maximize long-term average profit	ϵ−greedy	Non Apply	Undeclared			Simulation (Undeclared tool)
[44]	DQN	RAN	Enhance resource utilization and slices isolation	ϵ−greedy	Target NN, Online NN, replay memory, and mini-batch	Best effort, constant bit-rate, and minimum bit-rate			Simulation (Undeclared tool)
[45]	Q-learning DQN	RAN	Achieve a trade-off between the blocking and dropping probability of service requests	ϵ−greedy	Target NN and Online NN	Drop-sensitive and best-effort *			Simulation (3D Urban Macro—available [46,47])

*: non-5G/6G terminology is used for the use case or vertical application.

**Table 2:** Resource allocation based on RL.

Ref.	Algorithm	Focus	Optimization Objective	Explore-Exploit	Use Case/Vertical App	Training	Dataset	Development
[48]	Q-learning	RAN	Maximize resource utilization while meeting haptic communication performance requirement	ϵ−greedy	Haptic	Centralized		Simulation (Undeclared tool)
[49]	Q-learning, SARSA, Expected SARSA, & Monte Carlo	RAN	Guarantee efficient resource utilization while meeting low-latency requirements	ϵ−greedy	IoT	Centralized		Simulation (Undeclared tool)
[50]	Q-learning	RAN	Minimize end-to-end latency and maximize computing resource utilization	Undeclared	mMTC	Centralized		Simulation (5G K-SimNet)
[51]	Q-learning	RAN	Maximize profit and QoS satisfaction	ϵ−greedy	Undeclared	Centralized	Synthetic	Emulation (Mininet)
[52]	Multiagent PPO	E2E (RAN, TN, CN, Edge)	Maximize resource efficiency while meeting QoS	ϵ−greedy	Undeclared	Distributed		Emulation (Python-Pytorch)
[53,54]	Q-learning	RAN	Maximize resource utilization	Softmax	V2X	Centralized		Simulation (MATLAB)
[55]	Monte Carlo & Q-learning	Edge	Maximize social welfare / Maximize power allocation	ϵ−greedy	Undeclared	Centralized		Simulation (Undeclared tool)
[56]	Q-learning	RAN	Optimize latency, energy consumption, and cost	Undeclared	mMTC	Centralized		Simulation (Undeclared tool)
[57]	Multiagent Q-learning	RAN	Maximize profit while meeting end-to-end delay	ϵ−greedy	Undeclared	Distributed		Simulation (Undeclared tool)

**Table 3:** Resource allocation based on DRL.

Ref.	Algorithm	Focus	Optimization Objective	Explore-Exploit	NN Structure	Use Case/Vertical App	Training	Dataset	Development
[58,59]	DDQN & Dueling DQN	RAN	Maximize long-term profit while meeting diverse multitenants’ service demands	ϵ−greedy	Target NN, Online NN, replay memory, and mini-batch	Utilities, automotive, and manufacturing	Centralized	Synthetic	Emulation (TensorFlow)
[60]	DQN	RAN	Maximize radio resource utilization while QoS satisfaction	ϵ−greedy	Target NN, Online NN, replay memory, and mini-batch	eMBB, uRLLC, mIoT	Centralized	Synthetic	Simulation (Undeclared tool)
[61]	DQN	E2E (RAN, TN, CN, Edge)	Optimize VNFs positioning while meeting SFC traffic variations	ϵ−greedy	FNN	eMBB	Centralized	Real-available [62]	Emulation (openAI gym)
[63]	DQN	Edge, RAN & TN	Optimize resource utilization at the edge network	ϵ−greedy	DNN, replay memory, and mini-batch	Internet of vehicles and smart cities	Centralized	Synthetic	Simulation (Undeclared tool)
[64]	Dueling GAN-DDQN	RAN	Maximize profit and resource utilization	ϵ−greedy	Target NN, Online NN, Discriminator NN, memory replay, and mini-batch	VoLTE *, Video, and uRLLC	Centralized	Synthetic	Simulation (Undeclared tool)
[65]	LSTM-A2C	RAN	Maximize spectral efficiency, SLA satisfaction ratio, and profit	Softmax	Policy RNN and Value RNN	VoLTE *, eMBB, and uRLLC	Centralized	Synthetic	Simulation (Undeclared tool)
[66]	Constrained DQN	RAN	Maximizing resource utilization and throughput during orchestration and network slice management under service constraints	Softmax	FNN	Video, VoLTE *, and uRLLC	Centralized	Synthetic	Simulation (Undeclared tool)
[67,68,69]	DDQN	RAN	Minimize number of allocated radio resource blocks while meeting diverse and dynamic slice performance requirements	ϵ−greedy	Ape-X and replay Memory	Undeclared	Centralized	Synthetic	Simulation (NS3)
[70]	DQN	E2E (RAN, TN, CN, Edge)	Maximize QoE satisfaction and resource utilization	ϵ−greedy	FNN	V2X	Centralized	Synthetic	Simulation (Undeclared tool)
[71]	DQN	RAN	Maximize long-term revenue while ensuring QoS satisfaction	ϵ−greedy	Target NN, Online NN, replay memory, and mini-batch	Bandwidth sensitive *	Centralized	Synthetic	Simulation (MATLAB)
[72]	DQN	CN	Maximize QoS satisfaction and minimize deployment costs while meeting bandwidth and computing resources	Undeclared	FNN	Bandwidth sensitive *	Centralized	Real-available [73]	Emulation (TensorFlow)
[74]	DQN & DDQN	RAN	Maximize spectral utilization and minimizing costs	ϵ−greedy	Target NN, Oline NN, and replay memory	Elastic and real-time	Centralized	Synthetic	Simulation (Undeclared tool)
[75,76]	DQN	RAN	Maximize QoE satisfaction and resource utilization	ϵ−greedy	Target NN, Online NN, replay memory, and mini-batch	Delay constrained, rate constrained, rate and delay constrained, and rate and delay nonconstrained *	Centralized	Synthetic	Simulation (MATLAB)
[77]	DQN	Edge	Maximize resource utilization and QoS satisfaction	ϵ−greedy	Target NN, Online NN, replay memory, and mini-batch	Bit rate sensitive *	Centralized	Synthetic	Emulation (TensorFlow)
[78]	Variation of Actor-Critic	RAN	Maximize the total throughput over the time	Gaussian distribution	Policy NN and Value NN, replay memory, and mini-batch	Undeclared	Centralized	Synthetic	Simulation (Undeclared tool)
[79]	DQN	RAN	Maximize the data rate for eMBB and URLLC	ϵ−greedy	Online NN, Target NN, replay memory, and mini-batch	eMBB, and uRLLC	Distributed	Synthetic	Simulation (PyTorch)

*: non-5G/6G terminology is used for the use case or vertical application.

**Table 4:** Resource orchestration based on DRL.

Ref.	Algorithm	Focus	Optimization Objective	Explore-Exploit	NN Structure	Training	Dataset	Environment
[80]	DDPG	CN and Edge	Optimize placement of VNFs and service routing paths while addressing the enormous number of real-time traffic requests	Gaussian noise	Target NN and Online NN	Centralized	Synthetic	Emulation (TensorFlow)
[81]	DDQN	RAN	Maximize the expected long-term needs of tenants	ϵ−greedy	Target NN, Online NN, replay memory, and mini-batch	Distributed	Synthetic	Emulation (TensorFlow)
[82]	Online DQN	CN	Making chain placement decisions across geo-distributed data centers while minimizing deployment costs	ϵ−greedy	LSTM	Centralized	Real-available [83]	Emulation (Google data center)
[84]	TD3	RAN	Reconfigure computing resources autonomously while minimizing latency, energy consumption, and deployment costs	Gaussian	Policy Network and Value Network	Centralized	Synthetic	Emulation (OpenAI gym)
[85]	DDPG	E2E (RAN, TN, CN, Edge)	Maximize resource utilization while meeting SLAs	Decay Gaussian	Target NN, Online NN, memory replay, and mini-batch	Centralized	Real-available [86]	Emulation (Open air interface and open daylight)
[87]	Decentralized DQN	E2E (RAN, TN, CN, Edge)	Maximize slices’ performance under networking and computing resources constraints	Decay Gaussian	Target and Online NNs with actor–critic and replay memory	Distributed	Real-available [88]	Emulation (Open air interface and open daylight)

**Table 5:** Resource scheduling based on RL and DRL.

Ref.	Algorithm	Focus	Optimization Objective	Explore-Exploit	NN Structure	Use Case / Vertical App	Training	Dataset	Environment
[89]	A3C	RAN	Maximize resource utilization while guaranteeing slices isolation	Gaussian	LSTM	Undeclared	Distributed		Emulation (TensorFlow)
[90,91]	Q-learning	CN & TN	Minimize SFC’s delay	ϵ−greedy	Undeclared	Delay and none delay sensitive *	Centralized		Simulation (Undeclared tool)
[92]	QV-learning, QV2-learning, QVMAX-learning, QVMAX2-learning	RAN	Minimize packet delay and packet drop rate	ϵ−greedy and Boltzmann	Distributed NNs	Undeclared	Centralized		Simulation (LTESim)
[93]	DQN	E2E (RAN, TN, CN, Edge)	Minimize SLA violations while maximizing physical nodes’ resource utilization	Softmax	CNN	eMBB, uRLLC, mMTC	Centralized	Synthetic	Emulation (Python-Theano)
[94]	Q-learning	CN & TN	Achieve adaptive and cost-effective SFC	ϵ−greedy	Undeclared	Undeclared	Centralized		Simulation (Java-based)
[95]	DQN	RAN	Minimize latency	ϵ−greedy	FNN	uRLLC	Centralized		Simulation (Undeclared tool)
[96]	DQN	RAN	Maximize the long-term QoE	Softmax	Target NN, Online NN, and replay memory	Video streaming	Centralized		Simulation (Undeclared tool)

*: non 5G/6G terminology is used for the use case or vertical application.

Network slices support the provisioning of 5G use cases defined by the International Telecommunications Union, each with specific performance requirements [97]. The 5G use cases are known as eMBB, mMTC, and uRLLC [98,99]. The eMBB refers to services demanding high data traffic and a bit rate of 20 Gbps and 100 Mbps for user experiences in urban zones. The mMTC covers services requiring the connectivity of a wide gamma of devices and simplifies operational processes for providing a long battery lifetime. The uRLLC comprises services needing ultrahigh reliability and extremely low latency [100]. Although 5G networks have been widely deployed since 2020 [101], 6G networks appear on the horizon. This appearance is to address the exponential growth of emerging telecommunications services demanding more ambitious performance requirements. Furthermore, 5G features cannot entirely support extremely demanding services such as remote surgery and immersive media.

**Figure 1:**
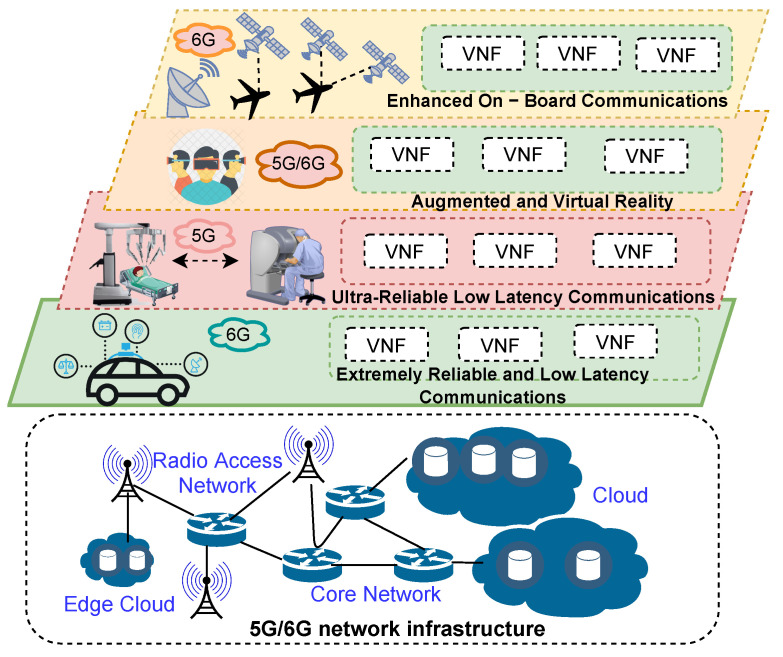
5G/6G network slices.

The 6G networks propose highly heterogeneous environments that are expected to provide global coverage, enhanced spectral/energy/cost efficiency, higher data rate (Tbps), 10 times lower latency, 100 times higher connection density, and full automation compared with 5G networks [102]. Technologies such as novel air interface and transmission techniques and architectures based on the IoT-Edge-Cloud continuum are fundamental for accomplishing 6G [1]. Potential 6G use cases include: sEMBB embraces EMBB services demanding high QoE; umMTC comprises services needing a much more massive number of simultaneous connections per space than mMTC; mURLLC covers mission-critical services requiring high reliability, low latency, and high availability; MBRLLC includes classical URLLC and eMBB services; ERLLC comprises services that merge URLLC and mMTC demands. These use cases are essential to accomplish intelligent home systems, smart cities, mission-critical applications, self-driving cars, and remote surgeries [36,103]; notably, they require approaches capable of performing dynamic network slicing resource management. Figure 1 shows different 5G/6G end-to-end slices built on a shared network infrastructure.

NSL follows an architecture formed by the infrastructure layer, the network function layer, and the service layer [104]. The infrastructure layer represents all physical elements (involving RAN, CN, and the edge network) needed by slices and the functions for controlling, operating, maintaining, and managing them. The network function layer encapsulates all configurations and life cycle management functions of the service function chains (SFCs) needed to realize end-to-end services that fulfill use cases’ performance requirements. NFV and SDN are fundamental technologies in this layer [105]. The service layer comprises the vertical applications, business models, XLAs/SLAs, and performance requirements of network slices. Intelligent and efficient resource management is fundamental for accomplishing the layers mentioned.

**Figure 2:**
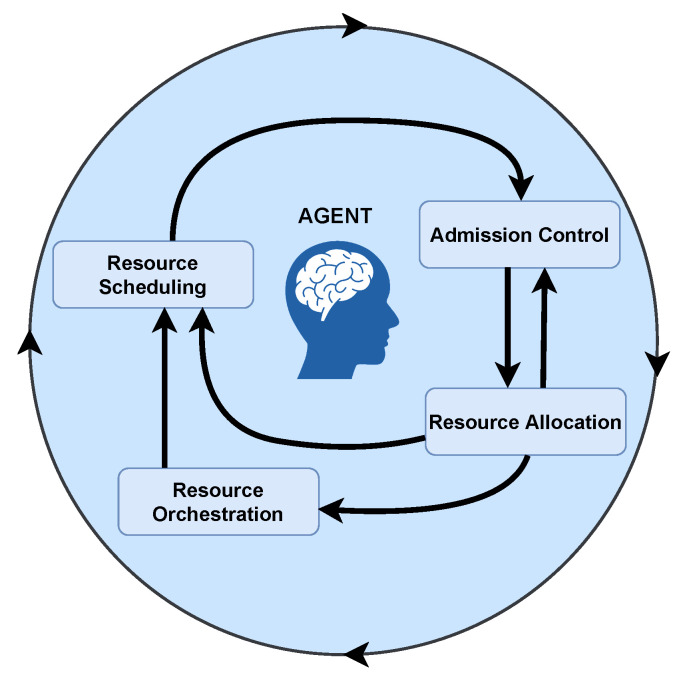
Resource management phases.

Resource management involves four phases (see Figure 2): admission control, resource allocation, resource scheduling, and resource orchestration. It is noteworthy that a phase can provide feedback to another one, and, as a result, they should operate coordinately. Admission control decides which slice requests coming from tenants (or a single one) can be accepted or not according to one or various network policies related to avoiding idle resources, increasing network providers’ revenue, and prioritizing services [10], for instance. Resource allocation quantifies the resources to assign per slice to fulfill, among others, the tenant demands, to meet QoS/QoE, and to maximize the long-term economic benefits of network providers [11]. Resource scheduling programs the time in which the network must allocate resources to each slice to, for instance, minimize the total execution and operation time of the network services, thus guaranteeing improved performance [12]. Resource orchestration mainly manages the service chains, their life cycle management, and the dynamic adjusting of assigned resources, taking into account, for example, performance requirements and network status [106,107]. The phases mentioned above are detailed in Section 3, Section 4, Section 5 and Section 6.

### 2.3. Deep Reinforcement Learning

DRL involves two fields of knowledge, namely RL and DL. RL is a machine learning (ML) approach appropriate for decision-making problems that need automatic handling based on trial and error. An RL agent periodically interacts with an environment by taking actions and receiving a reward (related to observations of the environment’s states) that indicates if the action was good or not [25,108]. RL can be understood as a Markov decision process (MDP) comprising a space of states *S*, a space of actions *A*, and an immediate reward function $R(s_{t},a_{t},s_{t+1})$ [109]. RL algorithms intend to find an optimal policy for maximizing the long-term reward in the environment by considering its states and the actions available per state.

RL algorithms can be model-based or model-free. A model-based RL algorithm [110] learns an optimal policy by having access to an environment’s model (a function able to predict the state, actions, and rewards) or obtaining it purely from experience. Model-free RL algorithms learn an optimal (stochastic or deterministic) policy (also known as on-policy algorithm) or optimal Q-value function (also known as off-policy algorithm) [26,27]. Actor–critic [20], state–action–reward–state–action (SARSA) [111], and proximal policy optimization (PPO) [112] exemplify on-policy RL algorithms. Q-learning [113] is the most popular off-policy RL algorithm. For further information about RL and its algorithms, we refer the reader to [20,114,115].

RL algorithms realize many interactions to achieve an optimal policy according to design requirements. The increasing number of iterations generates an expensive process due to the amount of information stored and the computational cost required. To overcome this challenge, ML proposes DRL that combines RL and DL to resolve high-dimensional and infinite-state problems [116]. DRL uses RL to train DNNs (e.g., FNNs and RNNs) that timely learn optimal policies [25,117]. Some of the most relevant DRL algorithms are deep Q-network (DQN—also known as deep Q-learning) [118,119], double DQN (DDQN) [120,121], deep Q-learning with prioritized experience replay (prioritized DQN) [122], Dueling DQN [123], and distributional DQN [124]. DRL and RL algorithms have been proposed in the 5G network in applications such as SDN routing [22], Internet of Things (IoT) [125], HetNets [126], and unmanned aerial vehicle (UAV) [127]. For further information about DRL and its algorithms, we refer the reader to [25,27,128].

The next Sections detail research papers that use RL and DRL to perform one or various resource management phases in NSL.

## 3. Admission Control

**Figure 3:**
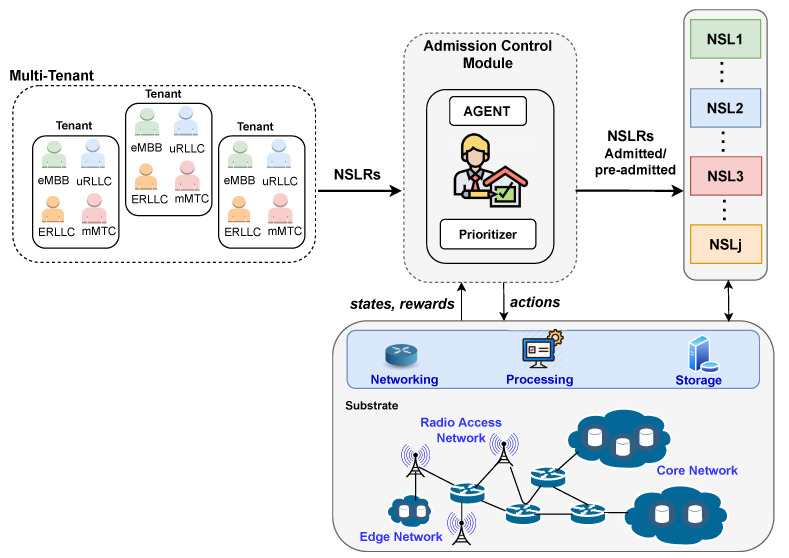
RL/DRL-based admission control architecture.

Recently, resource management literature has reported diverse admission control approaches centered on NSL. Those approaches have applied techniques such as dynamic programming [129,130], heuristics [131,132], and stochastic models [133,134,135,136] to accept slices in environments involving mainly a unique tenant. Figure 3 presents an admission control architecture using RL and DRL to make acceptance decisions in a multitenant environment. This architecture operates as follows. Tenants send diverse NSLRs (network slice requests) to the admission control module of 5G/6G use cases. The module decides the admission or preadmission (in this case, the allocation phase takes the final admission decision) of NSLRs by employing a RL/DRL agent and a prioritizer. The agent determines a normalized weight value for each 5G/6G use case. The prioritizer uses the agent’s outputs to sort the NSLRs and establish the order in which resources should be allocated in the corresponding phase. The weight values should lead to achieving a goal, for instance, obtaining the maximum profit. For example, in the raised example, the agent selects an action that, if taken, maximizes the profit. The agent learns to select actions that increase profit by considering the information on states and rewards from interaction with the environment by using, for instance, Q-learning or DQN. It is relevant to highlight that an RL-based admission control solution can be specified by defining its state space, action space, exploration and exploitation method, and reward function. In addition, the specification of a DRL-based admission control approach includes further defining its DNN structure. Note that these two points apply for specifying RL/DRL-based solutions of allocation, scheduling, and orchestration of resources.

### 3.1. Admission Based on RL and DRL

The following paragraphs review recent RL and DRL investigations in NSL or related technologies that perform admission control. Ref. [38] introduced an admission control algorithm called N3AC designed to maximize the price per time unit paid by inelastic and elastic network slices. Inelastic network slices were associated with uRLLC services. Elastic network slices were related to eMBB and mMTC services. N3AC trained two DNNs without a ground truth sequence (the proper sequence is unknown a priori); a DNN was used to estimate the revenue for each state when the action is to accept. The other DNN is for the rejecting action. In addition, N3AC modeled the state space as a three-sized tuple (Ne, Ni, k), where Ne and Ni are the numbers of inelastic and elastic slices, and k is the next event that indicates the arrival request or departure of a network slice. The space of actions was represented binary to admitting or rejecting new inelastic and elastic requests. The performance evaluation was performed in Keras/TensorFlow and included two elastic and two inelastic slices arriving by following a Poisson process. The time life of slices followed an exponential distribution.

Ref. [39] presented a SARSA (state-action-reward-state-action)-based cross-slice admission framework devised to maximize the operators’ revenue taking into account constraints related to communication, computing, and storage resources. The framework modeled the space of states considering demanded and deployed slices (best-effort and supporting QoS) and the resources available in the RAN and CN of 5G. The SARSA agent operated with a space of actions conceived as the number of slices to accept; it selected actions using the e-greedy method. The performance evaluation involved simulations in a nonspecified tool and six templates of slices not following the 5G use cases’ specific requirements. Refs. [40,41] proposed a RL-based admission control approach that uses DQN to maximize the providers’ total profit when dealing with low-profit services (e.g., on-demand media streaming and file transfer) and high-profit services (e.g., immersive media). The approach modeled the space of states regarding the available resources in the 5G-RAN. The DQN agent employed a space of actions involving holding time, service priority, and resources required. The performance evaluation included a custom-built Python-based event-driven simulator that used a networkX library for the graph representation and management of network resources and the Keras tool to implement the stochastic policy network.

Ref. [43] introduced an admission control mechanism based on R-learning [137] and Q-learning to maximize the long-term average profit in multidomain 5G-CNs. The mechanism modeled the space of states regarding the demand in the consumer and provider domains. The space of actions was defined as accepting service requests when the available capacity in the provider is greater than the total amount of demanded resources and rejecting in other cases. Ref. [44] proposed a DQN-based admission control approach for improving 5G radio resource management and enhancing isolation in three types of slices: best effort, constant bit-rate, and minimum bit-rate. The approach included two DNNs (online and target) in the DQN model to generate a learning policy that maximizes the cumulative reward. Online and target DNNs used an ReLU [138] with three layers of 50, 50, and 100 neurons and were employed to reduce errors in estimations. The DQN model defined the space of states in terms of key performance indicators (e.g., throughput, dropping rate, and admission rate). The space of actions was modeled considering control parameters for increasing or decreasing resources to the slices. Ref. [42] presented an admission control mechanism based on DQN with online and target DNNs for maximizing the providers’ revenue and minimizing the penalty cost caused by SLA violations in 5G-RAN. The DQN model used a space of states representing the number of slices requested of type eMBB, uRLLC, and mMTC and the type of the last slice request. The space of actions was modeled binary indicating whether the new arrival slice requests must be accepted or rejected.

Ref. [45] presented a Q-learning and DQN-based admission control approach for minimizing both the blocking probability of new requests and the dropping probability of admitted requests. The approach modeled the space of states regarding the resources used in the cells, the number of arriving requests, and the availability in neighboring cells. The space of actions was defined as blocking or accepting new requests from the users’ devices.

### 3.2. Remarks

Table 1 presents approaches using RL and DRL to perform admission control disjointly in RAN, TN, and CN of 5G. RL-based approaches (e.g., [43,45]) used SARSA and Q-learning to admit slices in RAN. They mainly consider maximizing the operator revenue and minimizing the total network cost as the optimization objective. The worst-case complexity of these algorithms is O(|S|×|A|) [39], where *S* and *A* are the sizes of the space of states and actions, respectively. RL-based approaches generally present scalability shortcomings when dealing with large state and action spaces. This shortcoming is relevant for slices of 5G/6G and beyond networks envisioned to support highly dynamic and complex services. DRL-based approaches [38,42,44,45] employed algorithms such as N3AC and DQN with one or two DNNs to admit slices in RAN while optimizing the operator revenue and network utilization as well as facing scalability issues. The worst-case complexity of these algorithms is O(|H|×|N|) [38], where *H* and *N* are the number of hidden layers and neurons, respectively.

Unfortunately, few works [39] have been developed to perform admission control involving end-to-end network slices. Indeed, most research has focused on proposing admission control approaches for 5G-RAN NSLs, neglecting the relevance of modeling aspects from the transport network and CN, for instance. In contrast, the TN, like the works [39,40,41], did by considering communication resources such as bandwidth and link optical backhaul and fronthaul. Since NSL is an end-to-end concept, novel admission control solutions in multitenant environments involving RAN, TN, CN, and edge networks are necessary to optimize revenue and utilization across the whole network. Since all cited works in Table 1 use centralized training, synthetic datasets, and nonperforming real deployments, it is essential to explore in-depth admission control based on decentralized, multiagent, and online RL/DRL to get a complete network view and cope with the dynamic of 5G/6G network slices. The use of datasets with real traces is essential to promote the deployment of approaches in real networks, which is, in turn, another imperative necessity. Furthermore, similar to [38,42], the admission control solutions should consider the QoS/QoE performance requirements of 5G/6G vertical applications in their models and operate coordinately with resource allocation approaches.

## 4. Resource Allocation

**Figure 4:**
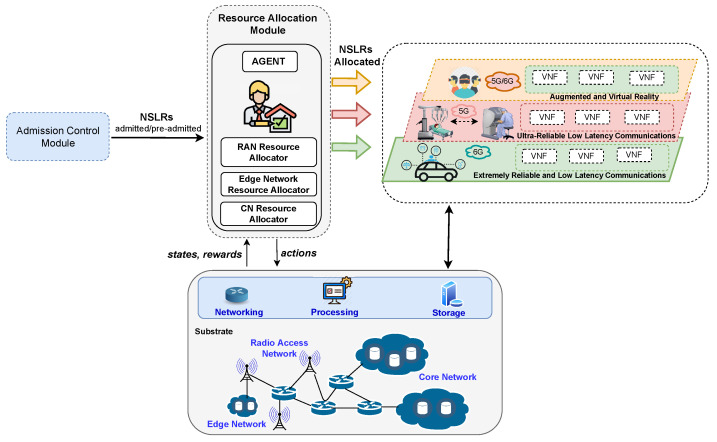
Resource allocation architecture using RL/DRL.

Lately, resource management literature has reported diverse NSL resource allocation approaches. Those approaches have applied techniques such as linear programming [139,140,141,142], (meta)heuristics [143,144,145,146], and game theory [10,147,148] to assign resources to slices in environments involving mainly a unique tenant. Figure 4 presents an architecture using RL and DRL to make resource allocation decisions in a multitenant environment. This architecture operates when the resource allocation module allocates RAN, CN, and edge network resources to preadmitted NSLRs (received from the admission control module) by employing a RL/DRL agent, a RAN resource allocator, an edge network resource allocator, and a CN resource allocator. The agent determines normalized weight values for each preadmitted NSLR belonging to a 5G/6G use case. Those values determine the allocation priority and the number of resources to assign per preadmitted NSLR and should lead to achieving a goal, for instance, obtain the minimum delay and maximum reliability. For example, in the raised example, the agent selects an action that, if taken, minimizes the delay and maximizes the reliability. The agent learns to select actions that decrease delay and increase reliability by considering the information on states and rewards from interaction with the environment by using, for example, policy gradient or DDQN. In the example, the allocators use the agent’s outputs to assign resources according to the priority and resources available in the network substrate.

### 4.1. Allocation Based on RL

The following paragraphs review modern investigations using RL to allocate resources in NSL or related technologies. Ref. [48] presented a radio resource allocation approach based on Q-learning and centered on 5G haptic communications for maximizing the utilization of scarce radio resources according to dynamism and the requirements of vertical haptic applications. The approach represented the space of states considering the allocated resources, application performance requirements, and resource utilization in each haptic vertical slice. The Q-learning agent modeled the space of actions in a binary way to denote the allocation or nonallocation of slices. The performance evaluation was performed in a nonspecified tool and conceived by a seven-cell hexagonal grid layout model with two vertical slices: a radio slice for the connectivity service to haptic communications and the other for human-to-human communications. The haptic devices were randomly distributed throughout the radio slice, while the users requesting the vertical applications followed a Poisson distribution. Ref. [49] presented a resource allocation framework based on various RL algorithms, such as Q-learning, SARSA, expected SARSA, and Monte Carlo, and devised to maximize the efficient utilization of resources in 5G Fog-RAN while guaranteeing the low-latency requirements of IoT applications. The framework modeled the space of states as resource (computing and processing) blocks occupied in fog nodes and IoT applications’ characteristics (latency, throughput, and channel capacity). The actions decided the appropriate layer (fog or cloud) to assign resources to provide IoT applications. The performance evaluation involved an IoT environment, including 10 applications from diverse domains (smart farming, smart retail, smart home, wearables, entertainment, smart grid, smart city, industrial Internet, autonomous vehicles, and connected health) with different latency requirements and profit features.

Ref. [50] introduced a Q-learning-based resource allocation method to minimize end-to-end latency and improve computing resource utilization in 5G Fog-RAN. The method modeled the space of states regarding the user requests, request arrival rate, percentage of allocated resources, percentage of unused allocated resources, minimum allocation requirements, and the maximum delay allowed. The Q-learning agent operated a space of actions defined to allocate or not compute resources. The method was evaluated in an open-source 5G K network simulator based on NS3 while openAI gym served to implement the Q-learning algorithm. In low-orbit satellite networks, Ref. [51] proposed a dynamic resource allocation approach based on Q-learning for maximizing the provider’s revenue (also known as system utility) and improving the users’ QoS satisfaction. The approach modeled the space of states considering the allocated and utilized resources per slice in a specific time *t*. The Q-learning agent used the actions to indicate whether the resource unit must be allocated (or not) for a particular user. The evaluation included two low-orbit satellite slices containing fixed radio resource pools. Mininet emulated the abovementioned slices.

Refs. [53,54] introduced an efficient resource allocation scheme based on Q-learning, focused on eMBB and vehicle-to-everything (V2X) services on 5G-RAN, and devised to maximize the overall resource utilization taking into account the services’ performance requirements and traffic dynamism. The scheme considered a space of states modeled as the number of resource blocks of cell bandwidth in the uplinks and downlinks. The actions were represented regarding allocation ratios of eMBB and V2X slices. Ref. [55] proposed an approach based on Monte Carlo [149] and Q-learning in an edge-computing and multitenant environment seeking to provide social welfare, meet QoS requirements, and maximize power resource allocation per network slice. The approach operated with a binary space of states where one indicated interference in the resource block assigned to a small cell base station of a particular tenant and zero the contrary. The action space was defined as the power level to allocate. Ref. [56] presented a Q-learning-based, dynamic, and autonomous computing resource allocation scheme intended to optimize the latency, energy consumption, and cost in 5G Fog-RAN. The scheme modeled the space of states as a vector comprising the allocated compute resource, average CPU utilization, and CPU reservation. The space of actions was defined regarding the resources to allocate at the Fog-RAN node level.

Ref. [52] presented a dynamic resource allocation framework based on PPO (proximal policy optimization) and intended to maximize resource efficiency while meeting QoS in end-to-end NSL in multilayer mobile edge computing environments. The framework modeled the space of states considering service type, the utilization of resources allocated in the edge nodes, and the ratio of offloaded workload in the edge–cloud continuum. The actions corresponded to tuning resources size (increased or decreased CPU and bandwidth) in a chosen node. Ref. [57] introduced a resource allocation framework based on a two-stage Q-learning algorithm for increasing operators’ revenue in a multitenant 5G network slicing environment. The first stage was devised to perform VNF mapping using a space of states based on the number of server nodes and computing resources available. The space of actions represented the association between a VNF and a physical server node. The second stage was conceived to carry out user association and power allocation, including a space of states associated with available radio resources and actions modeled as a set of vectors composed by the users and their corresponding power.

### 4.2. Allocation Based on DRL

The following paragraphs review modern investigations using DRL to allocate resources in NSL or related technologies. Refs. [58,59] presented a framework that allocates computing, storage, and radio resources to manufacturing services for maximizing the providers’ long-term incomes. The framework used DDQN and dueling DQN agents trained by the stochastic gradient descent (SGD) algorithm [150] and modeled their states considering requested resources, available computing resources, and connectivity capabilities of data centers responsible for storing virtualized radio resources. The agents operated with actions defined as the resources to assign per request. Using TensorFlow, the environment was emulated by creating three slice classes (i.e., utilities, automotive, and manufacturing) under different parameter settings. The slice requests followed a Poisson distribution.

Ref. [60] proposed a DQN-based strategy that allocates the radio and backhaul resources in a virtualized RAN to balance the QoS satisfaction and resource utilization of slices. The strategy considered the space of states as a vector involving the satisfaction ratio and the resources allocated to a slice. The DQN agent operated with actions modeled as percentages representing optimal resource provisioning. The evaluation involved an emulated mobile network with four classes of slices (enhanced-user equipment broadband, ultralow-latency communications, massive Internet of Things, and high-definition TV) following an exponential distribution. Ref. [61] proposed a resource allocation method based on a proprietary RL algorithm and a DNN to optimize the positioning of functions forming service function chains in metro-core optical networks. Four hidden layers formed the DNN with 100 nodes per layer. The method modeled the space of states regarding three layers (optical, IP/MPLS, and service slicing) and the space of actions as a decision state that indicates if one or more service function chains need reconfiguration. The emulation environment and algorithm were created using openAI gym; the proposed algorithm was trained using a mobile traffic dataset of the Milan urban area [62].

Ref. [66] introduced a resource allocation framework based on constrained DQN formed by a DNN (composed of two fully connected layers with 64 and 32 nodes) trained with various RL algorithms, and devised to meet performance requirements of video, VoLTE, and uRLLC slices in 5G-RAN. The framework modeled the space of states as the number of active users per service. The space of actions defined the bandwidth to allocate for each service. Ref. [64] introduced a resource allocation approach based on powered DDQN to meet SLAs as well as maximize resource utilization and provider revenue according to the dynamics of service requests on 5G-RAN. The approach used two generative adversarial networks (GANs) trained by the gradient descent (GD) algorithm [151] to minimize the difference between the estimated action–value distribution and the target action value distribution. Dueling GAN-DDQN represented the space of states as the service demands within a specific time window and modeled the space of actions as the bandwidth to assign to each slice. Refs. [67,68,69] presented an approach based on DQN that flexibly allocated resources on 5G-RAN to maximize network slice requirement satisfaction and improve resource blocks usage ratio. The approach modeled the space of states as the available radio resource blocks and the space of actions as allocation or not of resource blocks. Furthermore, the approach used the Ape-X method [152] to accelerate the learning by processing multiple DQN agents.

Ref. [70] introduced a two-tier resource allocation approach aimed to meet QoE requirements and achieve efficient utilization on 5G end-to-end slicing. The first tier proposed a dynamic resource optimization problem for allocating the radio resources under constraints on rate, power, and interference. The second tier employed DQN enhanced with two FNNs to allocate radio, edge, and cloud resources considering the slices’ arrival requests. The approach modeled the space of states representing available radio units and QoE satisfaction. The space of actions clustered remote radio heads to form access units intended to reduce intercell interference. Ref. [71] presented a DQN-based mechanism to allocate bandwidth and 5G-RAN resources to slices serving mobile, videos, and vehicle communications for increasing the long-term resource utilization and the revenue of virtual network providers. The DNN used ReLU as an activation function. The mechanism modeled the space of states as the requested bandwidth (arriving randomly) and the consumed energy. The space of actions was represented as the slice selected by the mobile virtual network operator to maximize profit.

Ref. [72] proposed a DQN-based approach to allocate bandwidth and virtual machines to services queued in a time window or during their arrival seeking optimizing delays and resource usage costs. The approach used a DNN activated by ReLu and designed the space of states as resource request arrivals and queueing levels from the last assignment. The actions represented binarily the operation of allocating bandwidth successfully. Ref. [74] introduced a resource allocation approach based on DQN and DDQN, focused on elastic and real-time slices, and conceived to maximize spectral efficiency utilization while reducing costs in 5G-RAN with many intelligent devices. The DNNs of DQN and DDQN used two hidden layers trained by the GD algorithm [153]. The approach modeled the space of states as the carrier power traffic assigned to each slice and the actions as a binary representation of bandwidth allocation per slice.

Refs. [75,76] proposed a DQN-based and dynamic framework that reserves and assigns unused bandwidth resources to virtualized RAN to maximize QoS satisfaction and resource utilization. The framework used a FNN with 4 neurons in the input layer, 2 hidden layers, and 20 neurons in the output layer. The space of states was defined considering the percentage of allocated virtual resources and the average resource utilization of each slice. The space of actions was modeled as the percentage for decreasing or increasing assigned resources. Ref. [77] presented a DQN-based resource management approach to reserve and allocate cache resources at the edge network for maximizing QoS satisfaction and network utilization to mobile virtual network operators. The DQN agent used a FNN with 4 and 11 neurons in the input and output layers and 2 hidden layers. The approach conceived the space of states regarding resource utilization, QoS satisfaction, reserved resources per slice, and allocated cache resource. The space of actions was defined to increase or decrease resources to cache slices. Ref. [65] introduced a bandwidth allocation strategy that uses a LSTM-based advantage actor–critic (A2C) [154] algorithm (i.e., it combines policy-based and value-based RL techniques) to maximize spectral efficiency, SLA satisfaction ratio, and profit in RAN. The LSTM-based A2C agent used Softmax [155] as activation function in the output layer. The strategy modeled the space of states as the number of slice arrival requests within a specific time window and the space of actions as the bandwidth to allocate to each slice.

Ref. [63] presented a DQN-based solution for allocating Internet vehicular and smart city applications. The DQN model included a fully connected DNN with an input layer, two hidden layers activated through ReLU, and an output layer activated through a linear activation function. The DQN agent operated with a space of states based on the number of resource blocks used at time *t*. The actions employed by the DQN agent allow for deciding if the user requests with heterogeneous latency demands and diverse computing loads must run at the cloudified RAN or edge network. Ref. [78] used a constrained discrete-continuous soft actor–critic algorithm to maximize the throughput in an environment with a discrete channel and continuous energy-harvesting time division. This actor–critic variation modeled the space of states considering the channel, battery and queue state. Furthermore, it represented its actions regarding the subchannel allocation and the harvesting time duration. Ref. [79] proposed a resource allocation mechanism that uses multiagents DQN and an SDN controller to allocate radio resources to uRLLC and eMBB to maximize the data rate. The DQN agents represented the space of states regarding the set of end-users, preallocated radio resource blocks, the channel gain, the minimum data rate, and the maximum delay. The agents operated with actions intended to assign the preallocated resource blocks to the end-users and request additional blocks from other agents.

### 4.3. Remarks

Table 2 and Table 3 present approaches using RL and DRL, respectively, to perform resource allocation mainly and separately in RAN and fog/edge network. RL-based approaches [48,49,50,51,52,53,54,55,56,57] used Q-learning, SARSA, expected SARSA, Monte Carlo, and actor–critic mainly to allocate radio or edge/fog resources to network slices efficiently, regarding operator revenue maximization, QoS satisfaction, and resource (computing and networking) utilization. Similar to Q-learning and SARSA (including variations), the worst-case complexity of Monte Carlo is O(|S|∗|A|) showing a dependence on the size of spaces of states and actions [49]. DRL-based approaches [58,59,60,64,65,66,67,68,69,71,72,74,75,76,77,78] employed algorithms such as DQN with one or two NNs, DDQN, dueling DQN, and LSTM-based A2C to allocate resources to RAN and (fog) edge network slices while optimizing the operator revenue and network utilization, and deal with scalability issues faced by RL-based approaches. Particularly, in [58,59] the worst-case complexity of DDQN and dueling DQN is $O(|S{|}^{2})$, where *S* is the size of the space of states. Furthermore, as mentioned earlier, the worst-case complexity of DQN is O(|H|∗|N|), which depends on the number of hidden layers and their neurons [60].

Regrettably, few works (e.g., [52,61,63,70]) have been developed to allocate resources to end-to-end network slices. Indeed, the investigations primarily focused on proposing resource allocation approaches for RAN and (fog) edge networks, disregarding the importance of TN and CN when accomplishing NSL. As NSL is an end-to-end concept, novel allocation solutions in multitenant ecosystems should involve elements from RAN, CN, edge networks, and TN (like [61,63] did by considering the availability of optical resources as wavelengths and links). They need to optimize revenue and utilization across the entire network and meet the QoS/QoE performance requirements demanded by 5G/6G use cases. Furthermore, the solutions should operate jointly with admission control approaches. Since almost all cited works in Table 2 and Table 3 use centralized training and synthetic datasets, it is needed to investigate the limitations and advantages of using multiagent (like [79] did) and online RL/DRL techniques to allocate resources to slices of multiple tenants dynamically. In addition, it is crucial to test the existing approaches with real datasets (such as in [61,72]) and networks (no work uses an actual deployment) to corroborate their practicability.

## 5. Resource Orchestration

**Figure 5:**
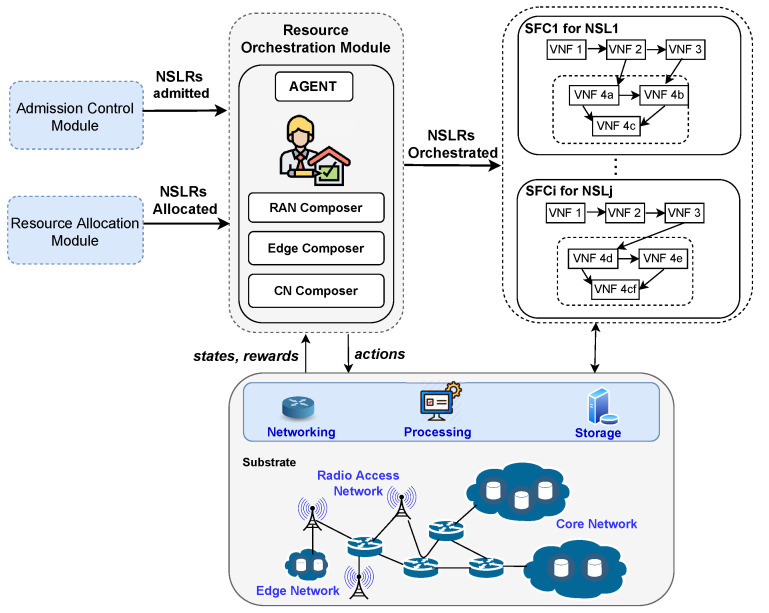
Resource orchestration architecture using RL/DRL.

In recent years, resource management literature has reported approaches that orchestrate resources in NSL by using techniques such as optimization [156,157,158,159] and (meta)heuris- tics [160,161,162]. Figure 5 presents an architecture using RL and DRL agents to orchestrate network slices. In this architecture, the resource orchestrator module composes RAN, CN, and edge network resources to form an end-to-end slice that follows the structure of admitted and scheduled NSLRs by employing a RL/DRL agent, a RAN composer, an edge composer, and a CN composer. The agent determines normalized weight values for composing services according to NSLRs of 5G/6G use cases. These values determine the orchestration priority, the (re)composition of service chains in RAN, CN, and edge networks, and traffic paths between the elements forming the end-to-end service (network slice in operation). Moreover, those values should lead to achieving an optimization goal, for instance, maximizing network utilization while meeting XLA. In the raised example, the Agent selects an action that, if taken, permits meet XLA and maximize network utilization. The agent learns to select actions that increase network utilization to avoid resource waste and allow meeting experience requirements by considering the information on states and rewards from interaction with the environment by using, for example, TD3 or online DQN. In the example, the composers use the values defined by the agent to build up and manage the life cycle of end-to-end network slices.

### 5.1. Orchestration Based on DRL

The following paragraphs review recent DRL investigations to perform resource orchestration in NSL or related technologies. Ref. [80] proposed a framework based on DQN, policy gradient, and actor–critic to orchestrate service function chains dynamically. The framework determines the placement of VNFs (cloud or edge) that form the service chains and the paths to connect them while guaranteeing real-time traffic requests. The DQN agent considered the space of states as the traffic flow rate of services and the status of VNFs. The framework defined the space of actions regarding the number of VNFs to activate and traffic flow to schedule. The performance evaluation included heterogeneous NFV/MEC-enabled IoT network scenarios emulated with the networkX tool. The authors used the scenarios to create a synthetic dataset helpful to train the framework’s algorithms in the TensorFlow framework. Ref. [81] introduced a DQN-based cross-slice resource orchestration approach to improve performance and maximize the expected long-term revenue in RAN slicing where multiple tenants compete for channel resources. The DQN agent used two DNNs with 2 hidden layers of 16 neurons; the Tanh function [163] was employed to activate the output layer. The approach defined the space of states as a tuple comprising data about mobile users (status and location) and arriving packets during a scheduling slot. The space of actions was modeled as the wireless radio resources to allocate to each tenant. The approach proposed was emulated using TensorFlow and trained with 5000 episodes. The environment comprised a physical RAN with 4 base stations covering 400 locations each. The requests raised by the mobile users followed a Poisson distribution.

Ref. [82] introduced a framework that scales and places service chains (composed of VNFs) seeking to achieve lower system costs in 5G networks. The framework used Online DRL extended with an actor–critic method that employed two LSTMs activated by ReLU. The online DRL agent operated with a space of states modeled considering data about the VNFs deployed in previous and current times and upcoming flows from the traffic model. The DRL agent defined the space of actions as the placement of service chains to serve all flows in a time *t*. The authors evaluated their framework using real-world Web traffic obtained from Huawei Inc., Hong Kong, China. [83]. Eight Google locations comprised the data center network used in the actual evaluation. Ref. [84] proposed a multiobjective approach based on TD3 (i.e., is a model-free, online, off-policy DRL method [164]) to orchestrate the computing resources in small cells connected to a central unit while minimizing the latency, energy consumption, and VNF instantiation costs (maximize profit). The TD3 agent used two DNNs activated by ReLu and modeled the space of states as the number of requested services per slice, the computing resources allocated to each VNF in the service chains, and the number of instantiated VNFs. According to traffic fluctuation, the approach defined the actions to increase or decrease the computing resources assigned to each VNF. The approach proposed used six deep neural networks implemented in PyTorch. The performance evaluation was realized in openAI gym considering two-tenant scenarios with different latency and CPU constraints requirements. The UE packet arrival followed a Poisson distribution.

Ref. [85] introduced a resources orchestration approach based on a constraint-aware online DRL algorithm devised to optimize resource utilization while meeting SLAs in end-to-end network slices. The DRL agent used two DNNs activated by Leaky ReLU [165]. The approach modeled the space of states considering the average traffic of slices, the number of users waiting in the queue, and the slice performance in the last time slot. The space of actions was defined regarding the resources (i.e., uplink and downlink physical resource blocks in RAN, bandwidth in the transport network, and computing resources in edge servers) to assign to each slice at a time slot. Ref. [87] also presented a decentralized approach based on deep deterministic policy gradient (DDPG integrates DQN and actor–critic) to efficiently orchestrate end-to-end resources regarding overhead and delay minimization while considering SLA violations and resources limitation in edge slices. The coordinator agent managed the resource orchestration policies and multiple orchestration agents. The decentralized agents estimated the resource demands of network slices and allocated the orchestrated resources locally. All agents operated with a space of states modeled regarding the status of network slices in queue and performance information provided by the coordinator and orchestration agents to estimate the resource demanded by network slices and orchestrate resources. The action space was defined as the resources to assign to network slices in the base stations and edge servers.

### 5.2. Remarks

Table 4 shows approaches successfully applying DRL to perform resource orchestration in NFV-based CN [80,82] and RAN [81,84]. Those DRL-based approaches used algorithms such as DQN, DDPG, online DQN, and TD3 to maximize provider revenue and decrease deployment costs while meeting performance metrics such as latency and energy consumption. The worst-case complexity of these algorithms is O(HN) and depends on *H* (number of hidden layers) and *N* (number of neurons). Further approaches considering a broader set of QoS and QoE performance metrics are needed.

Unlike the works cited above, Refs. [85,87] orchestrated resources in NSL from an end-to-end perspective. It is noteworthy that those works emulated the transport network with OpenDayLight and managed the resource allocation as a function of the available bandwidth to connect RAN, CN, and edge networks. Nonetheless, more investigations in multitenant environments involving RAN, CN, TN, and edge networks are needed to turn 5G/6G network slices into a reality. It is also necessary to provide NSL orchestration solutions for vertical application domains such as UAV, IoT, and tactile Internet. Moreover, providers need multiagent and noncentralized RL/DRL solutions to orchestrate slice resources to multiple tenants using a global network view and provide resource management capabilities inside the slice (i.e., in-slice management). It is noteworthy that various orchestration approaches were tested with real datasets and emulated in controlled scenarios. Therefore, the next step to determine their practicability is to evaluate them in more complex and realistic testbeds.

## 6. Resource Scheduling

**Figure 6:**
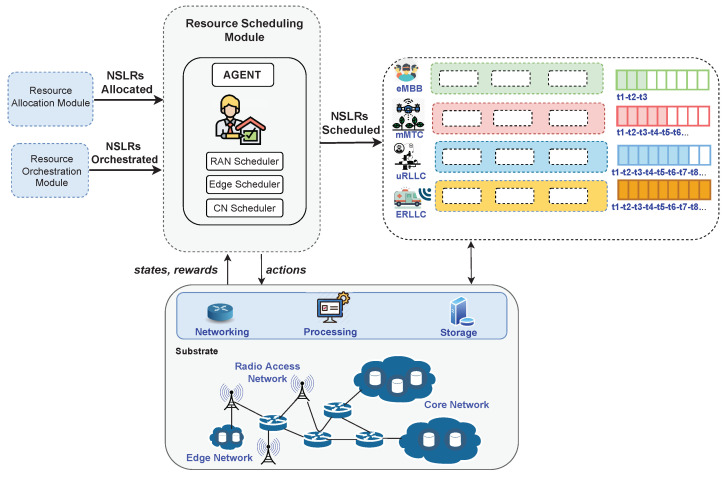
Resource scheduling architecture using RL/DRL.

Recently, resource management literature has reported diverse scheduling approaches centered on NSL. Those approaches have applied techniques such as (meta)heuristics [166,167], genetic algorithms [168,169], and job-shop problem [170,171] to program the execution time of elements composing a network slice. Figure 6 presents an architecture using RL and DRL to make resource scheduling decisions in a multitenant environment. In this architecture, the resource scheduling module schedules RAN, CN, and edge network resources to admitted NSLRs (after admission and allocation phases) by employing a RL/DRL agent, a RAN scheduler, an edge scheduler, and a CN sthe cheduler. The agent determines normalized weight values for each admitted NSLR belonging to a 5G/6G use case. Those values determine the scheduling priority, the time at which resources must be assigned, and the duration of each service offered by the admitted NSLR. Such values should lead to achieving a goal, for instance, meet performance requirements defined in SLA. In the raised example, the agent selects an action that, if taken, permits meeting the mentioned agreement. The agent learns to select actions that improve performance metrics included in SLA by considering the information on states and rewards from interaction with the environment by using, for example, A3C or dueling DQN. In the example, the schedulers use the values defined by the agent to program resource usage according to the priority, time and duration of slices, and resources available in the network substrate.

### 6.1. Scheduling Based on RL and DRL

The following paragraphs review latter investigations using RL and DRL to perform resource scheduling in NSL or related technologies. Ref. [89] presented an intelligent resource scheduling approach based on Asynchronous Advantage Actor-Critic (A3C) [172] (i.e., one of the most recent and powerful DRL algorithms) for improving resource utilization while guaranteeing isolation between slices in 5G-RAN. The approach operated with slices created on a substrate mobile network based on SDN and NFV. The A3C agent operated with a space of states represented as the set of users per slice and the assigned spectrum resources. The approach used a Gaussian probability distribution function to derive a stochastic policy for selecting actions modeled as resources programmed to each slice. The A3C was simulated in TensorFlow and trained with synthetic data traffic generated from Gaussian distribution. Refs. [90,91] presented a scheduling approach based on Q-learning to program the execution of VNFs composing a service function chain while minimizing delay. The approach considered delay as the difference between the end execution time of the first VNF and the end execution time of the last VNF belonging to the chain. The Q-learning agent employed a space of states modeled from the state of network function virtual infrastructure. The agent’s actions defined the VNF chosen for execution in a time *t*. The evaluation of the proposed approach considered a system composed of four NFV nodes and five network services delay-sensitive with different setting parameters. The packets of service arrived at the NFV nodes following a Poisson distribution.

Ref. [92] introduced a packet scheduler framework based on various RL algorithms and intended to minimize packet delay and packet drop rates in RAN slices sharing radio resources at each transmission time interval. The scheduler used QV-learning, QV2-learning, QVMAX-learning, and QVMAX2-learning [173,174] to achieve the optimal action–value function. The algorithms considered a space of states based on the quality indicator of channels, active users at each transmission time interval, arrival rates in data queues, and performance demands of network services. The framework modeled the state of actions as the number of resource blocks to allocate per transmission interval. The authors implemented the proposed framework in the LTE-Sim simulator with the RRM-Schedules C/C++ tool. For the evaluation, they used constant bit rate and variable bit rate to model the traffic of specific applications such as video, VoIP, FTP, and Web browsing. Furthermore, they generated constant traffic in random periods and variable traffic from a Pareto distribution. Ref. [94] proposed a Q-learning-based resource scheduling approach to achieve VNF chaining that is adaptive and cost-effective in 5G optical networks. The approach modeled the space of states regarding vCPUs used by each server node. The space of actions was represented as the physical server node selected for deploying VNFs in a time *t*.

Ref. [93] introduced an end-to-end NSL resource scheduling scheme based on DQN and intended to minimize the SLA violations of slices (guarantee performance and service reliability) and maximize resource utilization. The DQN agent learned to dynamically manage the resources of 5G network slices depending on the perceived demands by using a convolutional neural network (CNN) [175,176] composed of four convolutional layers; the output layer was activated by ReLU and Softmax functions. The scheme modeled the space of states regarding the number of allocated resources and the percentage of usage of all available resources scheduled for the slices. The space of actions was defined as the percentage in which the resources of each slice must be increased or decreased in a time *t*. Ref. [95] proposed a scheduling approach based on DQN and aimed to guarantee low-latency requirements and maximize data transmission downlink time in 5G-RAN when spectrum resources are insufficient. The DQN agent used a DNN formed by three and two neurons in the input and output (using ReLU) layers and three hidden layers. The approach considered the space of states as the total spectrum requirement, low-latency data delay constraints, and available spectrum. The space of actions was defined to assign 5G spectral resources during time intervals. Ref. [96] presented a DQN-based video stream scheduling solution to maximize the long-term QoE satisfaction of drones running on a 5G network. The DNN agent activated the output layer of DNN using the Softmax function and modeled the space of states regarding packet arrival rate, packet service rate, service slot duration, startup delay, traffic intensity, and packet arrival probability. The space of actions was defined to reconfigure the packet prefetching strategy and the startup delay.

### 6.2. Remarks

RL and DRL have been applied successfully to perform resource scheduling in NFV, RAN, and optical networks disjointly, as shown in Table 5. RL-based approaches [90,91,92,94] used algorithms such as Q-learning, QV2-learning, and QVMAX2-learning to schedule packets in service function chains as well as in radio and optical network slices to minimize cost, delay, and packet drops primordially. DRL-based approaches [89,95,96] employed algorithms such as DQN and A3C to schedule resources to 5G-RAN and UAV network slices while optimizing network utilization, SLA satisfaction, QoE meeting, and dealing with scalability issues of RL-based approaches. As the worst-case complexity of Q-learning and DQN was introduced in the early sections of this paper, here we show the complexity of the A3C algorithm [89]: $O(N*\left(1/N_{u}\right)*T_{c}*M*(\sum_{i=0}^{L_{a}}{u_{a}}^{i}*{u_{a}}^{\left(i+1\right)}+\sum_{j=0}^{L_{c}}{u_{c}}^{j}*{u_{c}}^{\left(j+1\right)}))$ where *N*: number of neurons, $N_{u}$: number of CPU threads used to train the algorithm, $T_{c}$: number of training steps, *M*: number of slices, *i* and *j*: number of units in the *i*th and *j*th layer of the DNN, and $u_{a}$ and $u_{c}$: number of units of the actor–critic network.

Unfortunately, few works (e.g., [90,91,94]) have investigated the scheduling of physical and virtual links to connect RAN, CN, and edge networks to guarantee the QoS of slices and providers’ profit or investigated resource scheduling NSL from an end-to-end perspective (like [93] did). Indeed, most research has focused on proposing scheduling approaches for 5G-RAN, disregarding the importance of TN, CN, and edge networks for realizing NSL. Since NSL is an end-to-end concept, more sophisticated solutions in multitenant environments involving elements from RAN, TN, CN, and edge networks are necessary to make 5G/6G network slices a reality. As in Table 5 only [89] used a distributed RL/DRL-based approach, novel solutions should consider multiagent, noncentralized, and online RL/DRL to handle the dynamism of 5G/6G networks and avoid making decisions with incomplete network views; the network overhead and consensus protocols are pivotal to assessing the feasibility of such solutions. Furthermore, the mentioned table corroborates and highlights the necessity to advance the RL/DRL-based scheduling approaches to operate with datasets containing real traces and evaluate them in real or testing networks. The need to obtain NSL solutions for other vertical application domains such as immersive media and remote surgery is also noteworthy.

## 7. Challenges and Future Research Directions

This section introduces some of the key unresolved challenges in network slicing resource management.

### 7.1. End-to-End and Coordinated Resource Management

As Table 1, Table 2, Table 3, Table 4 and Table 5 show, on the one hand, it is necessary to propose solutions that face the resource management problem in NSL from an E2E perspective. As most solutions are principally RAN-centered, they disregard one or more resources (mainly CN and edge network resources) needed to build network slices during their modeling. In this way, it is fundamental to investigate how to model, evaluate, and deploy E2E slices considering the three-dimensionality of 6G-RAN (nonstatic base stations based on UAV), fog-native architectures, and CNs supported on data center networks. On the other hand, collaborative solutions involving more than one resource management phase also are required to achieve E2E network slices. Notably, we consider that scheduling and orchestration phases in the RL/DRL-based network slicing resource management domain are still in their infancy in 5G/6G and beyond networks. We highlight that it is pivotal to study how to deploy RL/DRL-based solutions to operate E2E slices.

### 7.2. Multitenant and Vertical Oriented Resource Management

Considering 5G promotes realizing use cases such as uRLLC, mMTC, and eMBB and 6G advocates achieving sEMBB, umMTC, mURLLC, MBRLLC, and ERLLC, it is necessary to introduce new resource management solutions able to meet the requirements of such diversity of use cases. Indeed, Table 1, Table 2, Table 3, Table 4 and Table 5 show network slicing resource management solutions have not addressed 6G use cases to the best of our knowledge, opening the port for research on 6G resource management by using RL and DRL techniques. It is also remarkable that few solutions presented in Section 3, Section 4, Section 5 and Section 6 operate in multitenant environments and, worst, some of them do not use 5G or 6G terminology, resulting in a research gap to fulfill. Furthermore, novel resource management solutions will be needed to meet vertical applications’ QoE and QoS requirements such as remote surgery, immersive media, industrial IoT, and intelligent microgrids.

### 7.3. Incremental and Online Learning

Despite DRL, algorithms are practical for extremely high-dimensional application domains such as data center and 5G/6G networks. They usually operate with environments that remain unchanged during learning, as a result, they may present shortcomings when coping with dynamic environments where the reward function, state transition function, or state action spaces change over time. Incremental DRL [177,178] and online DRL [179] have been proposed recently for enhancing DRL. Incremental DRL algorithms can learn continuously, adapt their models without forgetting the learned earlier, and produce faster forecasting than traditional DRL algorithms operating with minibatches. Online DRL algorithms are incremental, operate in environments with restricted resources and hard run-time constraints, and have lifelong learning with limited data. As incremental DRL and online DRL are still in their infancy in the networking domain, we consider it worth investigating their benefits to a dynamic application domain such as 5G/6G network slicing resource management in depth. Several critical questions need an answer during those investigations: ① Which online/incremental DRL algorithms match resource management (or a particular phase)? ② What is the performance of these algorithms when solving resource management tasks? ③ How do we optimize existing online/incremental algorithms for resource management phases?

### 7.4. Distributed and Federated Learning

Distributed ML algorithms create accurate models using multiple servers usually containing datasets of around the same size with independent and identically distributed samples. These algorithms aim to improve the learning process regarding time, memory, and bandwidth. Federated learning is a particular distributed learning approach in which ML algorithms build accurate models from vast decentralized and heterogeneous datasets residing on resource-constrained devices (e.g., gateways, edge devices, smartphones, and autonomous vehicles). A federated learning process can be coordinated by a centralized node (e.g., a 5G/6G network data analytics function or an SDN controller) or collaboratively by distributed nodes (e.g., in-slice managers or programmable switches). Multiagent DRL [180] and federated DRL (FDRL) [181,182] have been proposed recently for enhancing DRL. It is worth exploring these DRL variations in 5G/6G networks since they could revolutionize the network-slicing resource management. Multiagent DRL and FDRL would learn deeply by interacting with the environment to meet XLAs and SLAs in multitenant and even multinetwork provider environments. Although some admission control and resource allocation approaches [183,184,185,186,187,188] have touched on these DRL variations, many research challenges have arisen (mainly related to scheduling and orchestration phases): ① Achieve an optimal trade-off between processing, memory, bandwidth, and accuracy requirements in the resource management solutions to facilitate their deployment in architectures based on microservices; ② Support concurrent and coordinated decisions in solutions involving more than one resource management phase in multitenant scenarios. ③ Build up open solutions and experimentation platforms to facilitate comparison and evaluation of FDRL-based solutions; ④ Combine online and federated learning to obtain resource management models that learn distributed and continuously when new data appear; ⑤ Provide security to FDRL, including securing the central coordinator, collaborator nodes, and updates of the shared model.

### 7.5. Explainable Models

RL and DRL have proven to successfully solve a range of sequential decision-making problems in networking and resource management. However, all approaches reviewed in this paper operate as black boxes (nontransparent and hard to interpret). They obfuscate their decision-making policy through complex Q-value functions or DNNs. Very few works concentrate on eXplainable RL (XRL) or eXplainable DRL (XDRL) that particularize the eXplainable AI concept (XAI). XAI intends to make AI-based solutions interpretable, manageable, and trustworthy [189]. XRL/XDRL is a relatively novel research field aimed at developing techniques to extract concepts from the RL/DRL agent’s (e.g., perception of the environment, intrinsic/extrinsic motivations/beliefs, Q-values) [190]. We consider XRL and XDRL to be essential to achieving real deployments and commercial success of RL/DRL-based solutions in 5G/6G resource management since operators and tenants can gain access to explanations and justifications of the outcomes given by XRL/XDRL solutions.

### 7.6. Practicability

RL/DRL solutions in networking have usually been evaluated in simulated scenarios, hindering their practical deployment. It is pivotal to evaluate those solutions, initially in emulated environments and, later, in real networks for commercial acceptance. Initial questions to address are ① How do novel ML advances test in network emulators? ② How can RL/DRL-based solutions be scaled from emulators to real-networks? As the real world is very different from simulations/emulations, it is necessary to answer additional questions. ③ How do RL/DRL-based solutions adapt to real dynamic data traces? ④ How do RL-DRL-based solutions scale in real dynamic networks? The raised questions constitute research gaps to make network resource management into a reality.

## 8. Conclusions

Recent years have witnessed explosive growth in using ML to solve networking issues. In particular, RL and DRL have been successfully applied in various networking areas. Specifically, this survey provides a comprehensive view of the applicability of RL and DRL techniques to perform resource management in 5G/6G network slicing. We reviewed representative research works and explored and discussed the feasibility and practicality of the proposed solutions in addressing admission control, resource allocation, resource scheduling, and resource orchestration challenges.

Future networks will have to support diverse QoE and QoS performance requirements from emerging use cases and vertical applications in multitenant environments. Although RL/DRL-based network slicing resource management solutions have shown promising results in simulated or (some few) emulated scenarios, their scalability and practicability need to be evaluated with the envisioned volume of data, ultrahigh number of devices, and applications (especially with real-time constraints) in small, medium, and large scale networks. On the other hand, current RL/DRL-based approaches for network slicing resource management offer mainly centralized and offline learning. To meet resource management on 5G/6G and beyond networks that are distributed in nature and operate with highly-dynamic data, existing RL/DRL approaches should be enhanced or re-architected to realize E2E network slices. This survey discussed the above issues along with several other challenges and opportunities. Our findings motivate the need for more research to advance the state-of-the-art seeking to achieve the vision of zero-touch network resource management.

## Data Availability

Not applicable.

## Abbreviations

A2C	Advantage Actor-Critic
A3C	Asynchronous Advantage Actor-Critic
ANN	Artificial Neural Network
CN	Core Network
CNN	Convolutional Neural Network
DDQN	Double Deep Q-Network
DL	Deep Learning
DNN	Deep Neural Network
DQN	Deep Q-Network
DRL	Deep Reinforcement Learning
E2E	End-to-End
eMBB	enhanced Mobile Broadband
ERLLC	Extremely Reliable and Low Latency Communications
FNN	Feed-forward Neural Network
GAN	Generative Adversarial Network
GAN-DDQN	Strategy Generative Adversarial Network Powered DDQN
GD	Gradient Descent
IoT	Internet of Things
HetNet	Heterogeneous Network
KPI	Key Performance Indicator
LSTM	Long Short-Term Memory
MBRLLC	Mobile Broadband Reliable Low Latency Communications
MDP	Markov Decision Process
MILP	Mixed Integer Linear Programming
MIoT	Massive Internet Of Things
ML	Machine Learning
mMTC	massive Machine Type Communication
mURLLC	massive ultraReliable Low Latency Communications
NFV	Network Functions Virtualization
NN	Neural Network
NSL	Network Slicing
NSLR	Network Slice Request
PPO	Proximal Policy Optimization
QoS	Quality of Service
QoE	Quality of Experience
RAN	Radio Access Network
ReLU	Rectified Linear Unit
RL	Reinforcement Learning
RNN	Recurrent Neural Network
SARSA	State-Action-Reward-State-Action
SDN	Software-Defined Networking
sEMBB	strengthened Enhanced Mobile Broadband
SFC	Service Function Chain
SGD	Stochastic Gradient Descent
SLA	Service Level Agreement
TD3	Twin Delayed Deep Deterministic Policy Gradient
TN	Transport Network
UAV	Unmanned Aerial Vehicle
umMTC	ultra Massive Machine-Type Communications
uRLLC	ultraReliable Low Latency Communication
V2X	Vehicle-to-Everything
VNF	Virtual Network Function
XLA	eXperience Level Agreement
